# Physicians’ perceptions of quality of care, professional autonomy, and job satisfaction in Canada, Norway, and the United States

**DOI:** 10.1186/1472-6963-13-516

**Published:** 2013-12-15

**Authors:** Reidar Tyssen, Karen S Palmer, Ingunn B Solberg, Edgar Voltmer, Erica Frank

**Affiliations:** 1 Department of Behavioural Sciences in Medicine, Institute of Basic Medical Sciences, Faculty of Medicine, University of Oslo, PO Box 1111, Blindern, Oslo NO-0317, Norway; 2 Faculty of Health Sciences and Faculty of Science, Simon Fraser University, Burnaby, BC, Canada; 3 Department of Health and Behavioural Sciences, Friedensau Adventist University, Möckern-Fridensau, Germany; 4 School of Population and Public Health, University of British Columbia, Vancouver, BC, Canada

## Abstract

**Background:**

We lack national and cross-national studies of physicians’ perceptions of quality of patient care, professional autonomy, and job satisfaction to inform clinicians and policymakers. This study aims to compare such perceptions in Canada, the United States (U.S.), and Norway.

**Methods:**

We analyzed data from large, nationwide, representative samples of physicians in Canada (n = 3,083), the U.S. (n = 6,628), and Norway (n = 638), examining demographics, job satisfaction, and professional autonomy.

**Results:**

Among U.S. physicians, 79% strongly agreed/agreed they could provide high quality patient care vs. only 46% of Canadian and 59% of Norwegian physicians. U.S. physicians also perceived more clinical autonomy and time with their patients, with differences remaining significant even after controlling for age, gender, and clinical hours. Women reported less adequate time, clinical freedom, and ability to provide high-quality care. Country differences were the strongest predictors for the professional autonomy variables. In all three countries, physicians’ perceptions of quality of care, clinical freedom, and time with patients influenced their overall job satisfaction. Fewer U.S. physicians reported their overall job satisfaction to be at-least-somewhat satisfied than did Norwegian and Canadian physicians.

**Conclusions:**

U.S. physicians perceived higher quality of patient care and greater professional autonomy, but somewhat lower job satisfaction than their colleagues in Norway and Canada. Differences in health care system financing and delivery might help explain this difference; Canada and Norway have more publicly-financed, not-for-profit health care delivery systems, vs. a more-privately-financed and profit-driven system in the U.S. None of these three highly-resourced countries, however, seem to have achieved an ideal health care system from the perspective of their physicians.

## Background

Although studies in some countries have compared patients’ opinions [1,2] about the influence of socioeconomic and health variables on health care systems, we lack cross-national studies comparing physicians’ opinions about practicing in their respective systems.

Our study compares physicians’ perception of quality of care, professional autonomy and job satisfaction between the health systems of Canada, Norway, and the U.S. This comparison is of interest for three reasons. First, doctors’ job-related perceptions (including their well-being) are reported to be key quality indicators for a nation’s health system [3,4]. Second, several studies link doctors’ satisfaction with the practice of medicine and patient satisfaction [5,6]. Third, given increased globalization [7] and migration among doctors for academic reasons or even due to financial crises [8-10], doctors should understand empirically what to expect of work-life in other countries.

In our comparison between the countries we controlled for age, because age has been associated with job satisfaction [11] and autonomy [12]. We also controlled for gender, as some studies have shown that women may be less satisfied with their autonomy than are men [13] and some studies have shown a gender difference regarding job satisfaction [11,14], though most studies do not [11]. Too many working hours and heavy workloads are related to decreased job satisfaction [15]; we therefore controlled for “hours in direct patient care”. Also, country differences between the United States (U.S.), Germany and Britain regarding time with each patient and differences in job satisfaction between medical specialties have been shown [16,17], we therefore control for this in our analysis. Whether a physician is self-employed or employed by others has been shown to be of importance to physicians’ satisfaction in the U.S. [18,19]. In Norway, most general practitioners and private practice specialists are remunerated by a combination of fee for service (60%) and capitation (40%), whereas most doctors working in hospitals are employed by the state-owned health trusts, complicating comparisons between the U.S. and Norway. We did not, therefore, include this variable in our analysis, nor did we include practice size because it was impossible to obtain these data for individual physicians in our samples, and because practice size was not a significant predictor of job satisfaction in another study that controlled for autonomy and other physician and practice characteristics (such as perceived time pressure) [20].

Professional control/autonomy is one of the most important predictors for job satisfaction [11,21]. Although job satisfaction and professional autonomy are closely linked to the quality of patient care and patient satisfaction [5,6,22], we lack studies of the interplay between professional autonomy and job satisfaction across differing health care systems. Definitions of professional autonomy vary, but all include freedom in clinical decision-making to provide high quality of care [21,22], adequate time for patients, and sustained relationships with patients [23], all factors independently associated with physicians’ career satisfaction [21,24,25]. As countries struggle to contain health care costs, many react with legislative initiatives hoping to simultaneously reduce costs and improve quality of care [26,27]. Physicians may feel these initiatives threaten professional autonomy.

National differences in the organization of health care service delivery structurally influence doctors’ working conditions [28]. Most obviously, in the U.S., health care delivery and financing are much more private, individual, and commodified than in the more public, collective, universal, and less-commercialized systems of Canada and Scandinavia. Although some variables have been compared among primary care doctors in the U.S., UK, and Germany [29], there are no comparative national studies of a more-representative sample of physicians’ opinions of their professional autonomy nor of the resultant quality of care they reportedly deliver. One comparison of primary care physicians from ten countries found that 68% of U.S. physicians were “satisfied/very satisfied” with practicing medicine, compared to 82% in Canada and 87% in Norway [30]. Correspondingly, more primary care physicians in the U.S. than in Canada and Norway felt a complete reform of their country’s health care system was necessary. We would therefore expect lower levels of satisfaction among the U.S. physicians in our comparison study of all specialties.

Comparisons across countries are challenging. We have used the best available data, despite different methods of data collection and different collection periods a few years apart. We analysed nationwide representative samples of physicians in all specialties. Based on the fact that the U.S. physicians work in a more private and individualized health care system we wanted to answer the following questions:

1) Is there a difference in the perception of autonomy and quality of care between physicians in the U.S., Canada and Norway when controlled for individual- and work-related factors?

2) Is the relationship between autonomy/quality of care and job satisfaction the same across the three countries when controlled for individual- and work-related factors?

## Methods

### Canadian physicians

The Canadian Physician Health Study (CPHS) was developed in 2007–8 in collaboration with Canadian medical organizations, primarily the Canadian Medical Association. We sent questionnaires to 8100 randomly selected Canadian physicians, excluding residents and retired physicians. The questionnaire and its distribution have been described in detail elsewhere [31]. The response rate was 41% (n = 3215/8100).

### Norwegian physicians

The Norwegian sample is from the Longitudinal Study of Norwegian Medical Students and Doctors (NORDOC). This postal survey originally included all medical students (N = 421) who began their studies at the four Norwegian universities in 1993, and all who graduated in 1993 and 1994 (N = 631). The present sample is from the fifth wave and 15-year follow-up of both cohorts in 2008. Procedures and cohorts have been described in detail elsewhere [32,33]. The response rate was 67% (n = 657/986).

### U.S. Physicians

The U.S. physician data base was the Community Tracking Study Physician Survey 2004–5 (CTS), representing direct patient-care physicians in the continental U.S. The sample included active, non-federal, office- and hospital-based physicians spending > =20 hours/week in direct patient care [16]. The survey was administered by computer-assisted telephone interview (CATI) and the weighted response rate was 52% (n = 6,628/12,648).

The CPHS received ethics approval from the University of British Columbia Institutional Review Board. The NORDOC survey was conducted according to the guidelines of the Ethical Committee for Medical Research and it was approved by the National Data Inspectorate of Norway. The CTS data were from round four of this longitudinal national survey of cross-sectional samples, where non-consenting doctors could choose to decline from the telephone interviews. There have been several publications from this survey; for details see Leigh et al. [16].

### Measures

We correlated the demographic variables of age, gender, specialty, and time in direct patient care, with two statements specific to professional autonomy and one about perceived quality of care that has been validated previously [21,23,24]: (1) “*I have adequate time to spend with my patients during a typical patient visit*” (adequate time); (2) *“I have the freedom to make clinical decisions that meet my patients’ needs”* (clinical freedom); and (3) *“It is possible to provide high quality care to all of my patients”* (high quality). These statements were presented with the exact same wording in all three national surveys using a five-point scale scored as 1 = 'strongly disagree'; 2 = 'disagree'; 3 = 'neither agree nor disagree'; 4 = 'agree'; and 5 = 'strongly agree'. The English statements were, according to convention, forward-backward translated into Norwegian by bilingual experts. The correlations between the three items in the respective country samples were in the range 0.26 to 0.53, and reliability analyses did not justify making an index of them (alphas <0.70). In addition, the items have been shown to represent separate and independent factors and “constructs” related to career satisfaction in both primary care and specialist U.S. physicians [21]. We therefore chose to analyse the three items as separate variables.

We also included a fourth question about overall job satisfaction : (4) *“On the whole, how satisfied are you with your job?”* (job satisfaction), presented in a five-category scale in the U.S. survey (using “career” instead of “job” satisfaction and scored as 1 'very dissatisfied'; 2 'somewhat dissatisfied'; 3 'neither satisfied nor dissatisfied'; 4 'somewhat satisfied'; 5 'very satisfied'); a four-category scale in the Canadian survey (1 'very satisfied'; 2 'somewhat satisfied'; 3 'somewhat dissatisfied'; 4 'very dissatisfied'); and a seven-point Likert Scale in the Norwegian survey (scored from 1 'extremely dissatisfied' to 7 'extremely satisfied'). Since the number of categories differed in each country, this variable could not easily be compared across the countries. Thus, we chose to run separate regressions on this variable for each country.

### Statistical analysis

Data analyses were conducted with SPSS for Windows Version 15.0. For continuous variables, data were analyzed using two tailed t-tests and univariate or multivariate analyses of variance (ANOVA) in a general linear model. Normality, linearity and homogeneity of variance were analyzed using UNIANOVA. Two models of linear regression determined the influence of independent variables such as age, gender, and hours in direct patient care on job satisfaction and each of the three professional autonomy variables (forced entry with cut-off scores of p < 0.05 for inclusion and p > 0.10 for exclusion). In order to validate the linear regressions we also performed logistic regressions on physicians’ perceptions of autonomy and quality of care.

## Results

### Sample description

Table 1 summarizes the main demographics and characteristics for the three national samples. Although 72% of U.S. and 63% of Canadian physicians were male, more Norwegian doctors (58%) were female. Canadian physicians (CAN) were older than the U.S. physicians and considerably older than Norwegian (NOR) physicians, most of whom were 35–44. More Canadians were Family/General Practitioners (40% CAN, 22% U.S., 23% NOR). U.S. and Canadian physicians most typically worked 40–49 hours/week in direct patient care (30% CAN, 31% U.S., 7.3% NOR); in Norway most worked 1–29 hours/week (59%) or 30–39 hours/week (33%) in direct patient care, and substantially more U.S. physicians (37.8%) worked ≥50 hours/week in direct patient care. Differences in age, gender, and hours in direct patient care were highly significant (p < 0.001).

**Table 1:** Demographics of the national samples

	**CPHS - Canada (N = 3213)**	**CTS - U.S. (N = 6,628)**	**NORDOC - Norway (N = 657)**
	**% (n)**	**% (n)**	**% (n)**
**Age**			
<35	8.0 (256)	5.9 (393)	5.7 (37)
35-44	23.4 (747)	30.6 (2030)	84.9 (552)
45-54	31.7 (1,014)	34.2 (2,267)	8.9 (58)
55-64	25.0 (799)	20.6 (1,367)	0.2 (1)
≥ 65	12.0 (383)	8.6 (571)	0.3 (2)
**Male**	63.0 (2,001)	72.1 (4,777)	41.8 (272)
**Female**	37.0 (1,174)	27.9 (1,851)	58.2 (379)
**Specialty**			
Internal medicine	4.6 (144)	16.2 (1,071)	7.2 (47)
Family/general practice	40.3 (1,267)	21.5 (1,427)	23.1 (152)
Pediatrics	4.4 (140)	12.0 (793)	5.3 (35)
Medical specialties	-	25.3 (1,674)	27.7 (182)
Surgical specialties	8.9 (280)	14.2 (941)	12.9 (85)
Psychiatry	7.5 (236)	5.5 (367)	10.5 (69)
ObGyn	3.6 (112)	5.4 (355)	3.7 (24)
Others	30.8 (968)		9.6 (63)
**Direct patient care hours**			
1-29	21.4 (664)	11.9 (789)	58.7 (384)
30-39	24.1 (747)	19.6 (1302)	32.7 (194)
40-49	30.1 (933)	30.7 (2032)	7.3 (43)
50-59	15.5 (479)	20.3 (1347)	1.0 (6)
60-69	7.0 (216)	10.5 (697)	0.2 (1)
>70	1.9 (60)	7.0 (461)	0.2 (1)

### Physicians’ perceptions of professional autonomy and quality of care

A much larger proportion of U.S. physicians as compared to Canadian or Norwegian physicians strongly agreed with these three statements (Table 2): “*I have adequate time to spend with my patients during a typical patient visit*” (adequate time: U.S. 29%, CAN 7%, NOR 7%); and “*I have the freedom to make clinical decisions that meet my patients’ needs”* (clinical freedom: U.S. 55%, CAN 10%, NOR 12%), and “*It is possible to provide high quality care to all of my patients*” (high quality care: U.S. 44%, CAN 5%, NOR 9%). After combining “strongly agree” and “agree”, inter-country differences diminished but were still very strong. The differences between the samples in these three statements (data not shown) were highly significant (F (6, 20) = 225.45, p < 0.001, η^2^ = 0.062), with a medium effect size (max η^2^ = 0.11 for high quality care): M(SD) adequate time CAN 3.06(1.13), U.S. 3.55(1.36), NOR 3.26(1.04); clinical freedom CAN 3.71(0.82), U.S. 4.28(1.04), NOR 3.78(0.74); high quality care CAN 3.14(1.05), U.S. 4.00(1.21), NOR 3.49(0.91). In *post hoc* testing, only the difference in clinical freedom between CAN and NOR failed to reach significance. Results did not significantly change in the CAN and NOR samples after adjustment for age, gender, specialty, when examining only those 35–44 years of age, or when excluding physicians with less than 20 hours/week of work in direct patient care.

**Table 2:** Physicians’ perceptions of professional autonomy, high quality of care, and job satisfaction in the three national samples (percentages in each category)

**Physicians’ perceptions**	**CPHS - Canada**			**CTS - U.S.**			**NORDOC - Norway**		
	**Total**	**Female**	**Male**	**Total**	**Female**	**Male**	**Total**	**Female**	**Male**
	**(n = 3213)**	**(n = 1174)**	**(n = 2001)**	**(N = 6628)**	**(n = 1851)**	**(n = 4777)**	**(N = 657)**	**(n = 378)**	**(n = 272)**
**Adequate time**									
Strongly agree	7	8	6	29	26	30	7	7	8
Agree	39	37	40	38	36	39	44	43	45
Neither agree nor disagree	18	18	18	2	2	2	21	20	20
Disagree	28	29	28	21	21	21	24	25	22
Strongly disagree	9	9	9	10	14	8	5	5	4
**Clinical freedom**									
Strongly agree	10	10	11	55	52	56	12	10	14
Agree	62	62	62	33	35	32	61	60	63
Neither agree nor disagree	17	18	17	2	1	2	21	22	19
Disagree	10	10	9	8	10	8	6	8	4
Strongly disagree	1	1	1	3	3	3	0	0	0
**High quality care**									
Strongly agree	5	4	6	44	40	46	9	7	12
Agree	41	39	42	35	38	34	50	48	53
Neither agree nor disagree	22	24	21	3	3	3	24	26	20
Disagree	26	29	24	13	14	13	17	18	14
Strongly disagree	6	5	6	5	6	5	1	1	1
**Job satisfaction**^**a**^									
Extremely satisfied	-	-	-	-	-	-	22	22	21
Very satisfied	46	42	47	42	42	41	44	43	45
Somewhat satisfied	44	48	42	42	42	42	24	24	23
Neither satisfied/dissatisfied	-	-	-	1	2	1	8	8	7
Somewhat dissatisfied	9	9	9	11	11	11	2	1	3
Very dissatisfied	2	1	2	4	3	4	1	1	1
Extremely dissatisfied	-	-	-	-	-	-	1	0	1

^a^CPHS four-category, CTS five-category, NORDOC seven-point Likert scale.

In three models (Table 3) we used perceptions of having adequate time, freedom of clinical decision-making, and providing high quality of care as the dependent variables. When adjusted for age, gender and hours in direct patient care the differences in country means accounted for the largest part of the explained variance (in total 6-11%), with higher scores among U.S. physicians as compared to Canadian and Norwegian physicians. Women had lower scores than men on each of the perceptions. The variables for age and hours in direct patient care were not completely normally distributed. Table 3 (i.e. the simple linear regression analyses) should be interpreted with this in mind. We also performed logistic regression analyses revealing the same significant predictor variables, thus strengthening our findings.

**Table 3:** Regression analysis (multivariate): variables predicting adequate time, freedom of clinical decisions, and high quality of care (five-point scale, 1 = strongly disagree, 5 = strongly agree)

	**Adequate time**			**Clinical freedom**			**High quality of care**		
	***Unst. B***	***95% CI***	***Adj. r***^***2***^ ***= 0.06***	***Unst. B***	***95% CI***	***Adj. r***^***2***^ ***= 0.07***	***Unst. B***	***95% CI***	***Adj. r***^***2***^ ***= 0.11***
**Age** (five age groups from 1 <35 to 5 > 65)	0.13*	0.11 to 0.16		0.00	-0.02 to 0.02		0.02	-0.01 to 0.04	
**Gender** (1 = male, 2 = female)	-0.13*	-0.18 to -0.07		-0.08*	-0.12 to -0.03		-0.13*	-0.18 to -0.08	
**Hours in direct patient care** (seven groups 1 <30 to 7 > 70)	-0.10*	-0.12 to -0.09		-0.05*	-0.07 to -0.04		-0.04*	-0.06 to -0.02	
**Country means**									
**U.S.** (reference)									
**Canada**	-0.55*	-0.60 to -0.49		-0.58*	-0.63 to -0.54		-0.88*	-0.93 to -0.83	
**Norway**	-0.30*	-0.41 to -0.18		-0.53*	-0.61 to -0.44		-0.51*	-0.61 to -0.40	

*p < 0.01.

### The impact of perceptions of quality of care and professional autonomy on job satisfaction

In separate regression analysis for each country (due to different number of categories), there were no gender differences in job satisfaction (Table 4). In Canada, older doctors were slightly more satisfied in their job (B = 0.04, p < 0.01), whereas in the U.S. younger doctors were more satisfied (B = -0.09, p < 0.01) and in Norway there was no age difference (probably due to the constrained age distribution of the sample). Hours in direct patient care were significantly related to job satisfaction in Canada and the U.S. (p < 0.01), and having adequate time with patients was significant (p < 0.01) in all three countries (Canada, B = 0.08, U.S., B = 0.11, Norway, B = 0.16). Having clinical freedom (Canada, B = 0.13, U.S., B = 0.20, Norway, B = 0.24) was a significant predictor (p < 0.01) in all three countries as was being able to provide high quality of care (Canada, B = 0.12, U.S., B = 0.13, Norway, B = 0.14). The hours in direct patient care and adequate time (block 2), and clinical freedom and high quality (block 3 and 4) accounted for the largest parts (11-15%) of the explained variance in the models. Among Norwegian and Canadian physicians, 90% rated their overall job satisfaction as at-least-somewhat satisfied, vs. 84% among the U.S. physicians (NOR vs US: Chi-square = 15.7, p < 0.001; CAN vs US: Chi-square = 64.1, p < 0.001).

**Table 4:** **Regression analysis (multivariate): variables predicting job satisfaction**^**a **^**for Canada, U.S., and Norway separately**

	**Canada**			**U.S.**			**Norway**		
	**Unst. B**	**95% CI**	**Adj. R**^**2 **^**= 0.14**	**Unst. B**	**95% CI**	**Adj. R**^**2 **^**= 0.15**	**Unst. B**	**95% CI**	**Adj. R**^**2**^ **= 0.12**
**Age** (five age groups from 1 < 35 to 5 >65)	0.04**	0.01 to 0.06		-0.09**	-0.12 to -0.07		0.10	-0.09 to 0.29	
**Gender** (1 = male, 2 = female)	0.00	-0.05 to 0.06		0.00	-0.06 to 0.06		0.14	-0.20 to 0.30	
**Hours in direct patient care**	-0.03**	-0.05 to -0.01		-0.06**	-0.08 to -0.04		0.00	-0.07 to 0.07	
**Adequate time** (five-point Likert scale from 1 strongly disagree to 5 strongly agree)	0.08**	0.05 to 0.10		0.11**	0.09 to 0.13		0.16**	0.08 to 0.24	
**Freedom for clinical decisions** (five-point Likert scale from 1 strongly disagree to 5 strongly agree)	0.13**	0.09 to 0.16		0.20**	0.17 to 0.23		0.24**	0.12 to 0.36	
**High quality of care** (five-point Likert scale from 1 strongly disagree to 5 strongly agree)	0.12**	0.09 to 0.15		0.13**	0.11 to 0.16		0.14**	0.04 to 0.23	

*p < 0.05,** p < 0.01.

^a^U.S. survey scored: 1 'very dissatisfied'; 2 'somewhat dissatisfied'; 3 'neither satisfied nor dissatisfied'; 4 'somewhat satisfied'; 5 'very satisfied'.

Canadian survey scored: 1 'very satisfied'; 2 'somewhat satisfied'; 3 'somewhat dissatisfied'; 4 'very dissatisfied'.

Norwegian survey scored (on a Likert Scale): 1 'extremely dissatisfied' to 7 'extremely satisfied'.

## Discussion

Although none of the physician samples were uniformly satisfied with their work experiences, U.S. physicians reported markedly higher perceptions of quality of care and professional autonomy than physicians from Canada and Norway (even when controlled for age, gender, and hours worked) but a lower rate of being at-least-somewhat satisfied with their jobs. In all three countries, physicians’ ability to provide high quality of care and having high professional autonomy were both related to higher overall job satisfaction.

We found that U.S. physicians are more likely to report having adequate time with their patients, a finding reported by U.S. primary care physicians in other studies. For example, despite reporting lower job satisfaction, U.S. primary care physicians reported higher time allocation for new patients and in particular shorter waiting times for a specialist appointment than in ten other countries including Canada and Norway [30,34]. The same held true in a comparison between the U.S., UK, and German primary care settings [29]. However, our study is the first to show this perception in nationwide samples that also include hospital specialists and, as such, our findings are more representative of all health services and settings. Notably, although U.S. physicians reported having adequate time with their patients, they also reported that they (more than UK and German physicians) wished for additional time with patients [29]. This relative dissatisfaction with the available time may help explain the discrepancy between job satisfaction and professional autonomy.

U.S. physicians also reported more freedom to make clinical decisions and in addition more possibility to provide high quality of care. All three variables of professional autonomy and quality of care were reported higher among the U.S. physicians, and this was found to be independent of age, gender, and hours in direct patient care, as shown in the multiple regression analyses in Table 3. This strengthens our finding of the differences in perceptions between the U.S. physicians and the Canadian and Norwegian physicians.

Some would argue that differing modes of data sampling may play a role in our study. The U.S. physicians were interviewed (computer-assisted) and they may be subject to so-called “social desirability bias” more than the others that were surveyed by mailed questionnaires [35]. But, as shown above, our findings concur with other cross-national studies among primary care physicians. Furthermore, the U.S. physicians express both more satisfaction (with professional autonomy) and dissatisfaction (with their work in general) than the others.

Consistent with our study of all physicians, other evidence shows that U.S. primary care physicians work longer hours per week than physicians in Canada and Norway [34]. Much of that time is occupied with non-clinical duties: 57% of U.S. physicians complained about time required for administrative tasks (vs. Canada 27%, Norway 13%) or for arranging care in cases of limited health care coverage (U.S. 48%, Canada 19%, Norway 17%). Time and related costs for interacting with health care administration was found to be much higher in U.S. physicians than in their Canadian colleagues [36].

Our results showed that in both the U.S. and Canada, hours in direct patient care were negatively related to physicians’ job satisfaction, but there was no such association in Norway. In Norway, working hours are highly regulated and weekly working hours rarely exceed 60 [37].

Canada and Norway have more publicly-financed, not-for-profit health care systems, vs. the more privately-financed and profit-driven system in the U.S. Among U.S. physicians, perceived autonomy may be associated with a perception of plenty, arising from relatively-abundant technology and access to care for well-insured patients or those wealthy enough to bypass the restrictive private for-profit insurance system altogether.

In contrast, and in spite of this perception of plenty, several factors may contribute to physician burnout, leading to the lower overall job satisfaction measured among U.S. physicians here and validated in other studies [30,34,38,39]. These may include problems in the U.S. with accessing care (especially for uninsured or underinsured patients) [40], changes in practice environment [41], rising inequity in access to care [42], values that are incongruent with the health care system [43], and discouraging preventable health outcomes [44] (like burgeoning obesity, adverse drug reactions, hospital errors, and relatively high infant mortality). Further research is needed to explain the higher perception of professional autonomy coupled with lower job satisfaction in U.S. physicians as compared to physicians in Canada and Norway.

Despite spending more as a percentage of GDP on health care than the OECD average, stable job satisfaction [45], and overall positive assessment of the health care system [34,45], 66% of Norwegian physicians reported distress due to waiting lists and to patient care impaired by time constraints [46]. More than half of Norwegian physicians (55%) also complained about time spent on administration and documentation.

Canadian physicians perceive longer waits for diagnostic procedures than do U.S. physicians, though physicians’ perceptions may not be consistent with the wait time evidence [47,48]. Patient surveys of waiting times show the U.S. ranks last among seven countries on dimensions of access to care [49], but there is scarce evidence on physician perception about waiting times in the U.S. In contrast, Canadian provinces measure and publicly report physician-specific waiting times for elective surgical procedures. Additionally, the perception of independence (even in the context of corporate and other controls over health care delivery) is a highly socially-valued condition in the U.S., whereas interdependence is more valued in the relatively collectivistic health care systems of Norway and Canada. All these variables could be among the underlying reasons for the lower scores of perceived professional autonomy, despite relatively high overall job satisfaction in both Norway and Canada.

Gender differences were consistent in the adjusted analyses of all three perceptions: women physicians reported lower perceived professional autonomy and quality of care. The reasons for this are uncertain, though we know female doctors tend to have better communication skills [50,51], and may be more sensitive to threats to both professional autonomy and quality of care in time-pressured work sites [38,46]. Previous review studies have shown the effect of professional autonomy on job satisfaction [11,15,52], which validate our findings. But there are fewer studies that show the relationship between doctors’ perception of quality of care and job satisfaction [22,53-55], so our findings from three different countries strengthen the notion that quality of care is important both for patients and for physicians’ job satisfaction.

### Limitations

First, this is a cross-sectional study and we cannot infer causality. Those who are most satisfied in their jobs may be those who report highest professional autonomy as well as the other way round. Measuring job satisfaction with a different number of categories in each sample complicates comparison between countries, though regressions have been done separately for each country. The lower response rates in the U.S. and Canadian samples are limitations, but they should not affect the associations in the regression models. We are also limited by some differences in sampling strategies. For example, the U.S. sample excluded physicians working less than 20 hours per week, though exclusion of these physicians in the Canadian and Norwegian samples did not change the significant differences; nor did the differences change after adjusting for age, gender, and specialty, or focusing the analysis on the younger age group (35–44 years) most prominent in the smaller Norwegian sample. There may be some effect from different modes of data collecting (interview versus survey), although this effect is likely modest, as described for above. The U.S. data were 3–4 years older than the Canadian and Norwegian ones. Nevertheless, there were no major health reforms over these years (2004–2008) in any of the countries that could influence our findings. There was relatively-limited explained variance in our regression models, and other possible explanatory variables that may influence job satisfaction or professional autonomy were not studied, such as one’s source of practice revenue, the administrative complexity of collecting reimbursement for services, physician perception of waiting times, and practice type and/or size.

## Conclusions

In this first cross-national comparison study, U.S. physicians reported much higher perceptions of quality of patient care and professional autonomy (including having adequate time with patients and freedom to make clinical decisions) compared to Canadian and Norwegian physicians, though somewhat lower job satisfaction. In all three countries quality of care and professional autonomy were related to overall job satisfaction, and women physicians reported lower rating for all three items. Further international comparative research is warranted to better-describe the constellation of factors affecting perceived quality of care, professional autonomy and job satisfaction in physicians around the world.

## Competing interests

The authors declare that they have no competing interests.

## Authors’ contributions

EF and RT conceived of the comparison study (EF and RT also designed and ran the CPHS and the NORDOC surveys, respectively). EV, RT and IBS performed the statistical analyses. EV and RT initiated the drafts, whereas also EF, IBS and KSP wrote and reviewed multiple drafts. All authors read and approved the final manuscript.

## Pre-publication history

The pre-publication history for this paper can be accessed here:

http://www.biomedcentral.com/1472-6963/13/516/prepub
